# No effect of preliminarily simulated cathodal HD-tDCS on the frontopolar cortex in the exploration-exploitation task

**DOI:** 10.1038/s41598-025-22016-z

**Published:** 2025-10-31

**Authors:** Viktor Timokhov, Andrey Timashkov, Oksana Zinchenko

**Affiliations:** 1 Zurich Center for Neuroeconomics, Department of Economics, University of Zurich, Zürich, Switzerland; 2 Centre for Cognition and Decision-Making, Institute for Cognitive Neuroscience, National Research University Higher School of Economics, Moscow, Russia

**Keywords:** Exploration-exploitation, tDCS, fNIRS, Modeling, Cognitive neuroscience, Computational neuroscience, Learning and memory, Reward

## Abstract

This study investigated the causal role of the human frontopolar cortex (FPC) in managing counterfactual strategies during decision-making under uncertainty. Using a combination of cathodal high-definition transcranial direct current stimulation (HD-tDCS) and computational modeling, we examined how cathodal stimulation, preliminarily simulated with SimNIBS, affects the retrieval of alternative strategies in the task that constantly provokes exploration-exploitation dilemmas. A comparison of resting-state recordings with functional near-infrared spectroscopy (fNIRS) before and after stimulation did not reveal any physiological effect of HD-tDCS. Furthermore, there was no significant difference in participants’ exploratory behavior between the sham and cathodal groups. These results indicate that the employed HD-tDCS protocol may not sufficiently influence FPC function, despite preliminary tDCS simulations with SimNIBS, calling for further refinement of the HD-tDCS protocol used in future research to elucidate the causal role of the FPC in decision-making under uncertainty.

**Supplementary Information:**

The online version contains supplementary material available at 10.1038/s41598-025-22016-z.

## Introduction

Decision-making under uncertainty often involves balancing the exploitation of an established behavioral strategy with the exploration of alternative ones. This so-called *“exploitation-exploration” dilemma* is fundamental for executive control and critical for efficient adaptive behavior in a volatile everyday environment. A basic solution to this dilemma can be achieved through reinforcement learning (RL), which converges on a behavioral strategy maximizing rewards when they depend only on external states and actions^1^. However, the open-endedness of the everyday environment implies a memory-like mechanism that stores previously learned and ongoing behaviors in the form of discrete entities, referred to as task sets. This feature implies an infinite number of dimensions, with which basic RL cannot deal. Besides, no physical system can represent such an infinite-dimensional space in parametric fashion^2^.

This infinite dimensionality problem can be addressed with mixtures of Dirichlet processes (MDP), which offer optimal mathematical solutions to infinite-dimensional problems^3^. Based on the MDP, a biologically plausible computational model of human reasoning (PROBE model) that resolves the “exploitation–exploration dilemma” was proposed^4^. Mathematically speaking, the PROBE model is based on reinforcement learning and Bayesian techniques and approximates mixtures of Dirichlet processes. Researchers have also described the neural basis of the PROBE model in the prefrontal cortex^5^. The neural implementation of this model includes two concurrent inferential tracks in the prefrontal cortex: the first makes probabilistic inferences about the *reliability of the ongoing strategy*, arbitrates between exploring new strategies and adjusting the current strategy, and comes from the ventromedial to the dorsomedial prefrontal cortex (vmPFC and dmPFC, respectively). Another track from the polar to the lateral prefrontal cortex (FPC and laPFC, respectively) makes probabilistic inferences about the *reliability of two or three alternative (or counterfactual) strategies* and arbitrates between exploring new strategies and exploiting alternative ones. Together, these tracks implement hypothesis testing for rejecting or accepting newly created strategies.

Koechlin (2014)^6^ traced the evolution of prefrontal executive function from rodents to humans and highlighted that the role of the polar prefrontal cortex, or frontopolar cortex (FPC), in monitoring counterfactual strategies is unique to humans. Using fMRI and the PROBE model, Donoso, Collins, and Koechlin (2014)^5^ demonstrated the crucial role of the FPC in monitoring counterfactual strategies during the task performance for human participants. Nonetheless, to better understand the relationship between behavior and neural signals, it is important to study both the correlation and causation between brain events and mental events^7,8^. Previous research on prefrontal executive function theory in humans, however, primarily relied on correlational methods^5^, where activity in the prefrontal cortex was linked to computational model predictions via fMRI. In more recent publications on this model, the exact neural mechanisms of the vmPFC-dmPFC track were shown via intracranial EEG, revealing predictive coding mechanism used for reward prediction^9^. Building on this evidence, the aim of the present research was to study the causal role of the human frontopolar cortex (FPC) in processing and storing information about alternative strategies and the efficiency of their retrieval. Based on earlier fMRI findings, we hypothesized that the inhibition of neural activity in the right FPC with cathodal high-definition transcranial direct current stimulation (HD-tDCS) would reduce the efficacy of alternative strategy retrieval and, consequently, promote more exploratory behavior compared to sham stimulation.

## Results

We employed a task specifically designed to elicit the resolution of exploitation-exploration dilemmas on each trial, previously used in studies of the PROBE model^4,5,9^. Participants had to learn combinations of three unique numbers that corresponded to four response buttons (one number per button and one button without any number, e.g., “1” for the “u” button, “3” for the “o” button, “5” for the “p” button, and nothing for the “i” button). On each trial, one of three numbers was presented, and participants had to press the corresponding button. After each trial, participants received feedback, either positive or negative. Thus, participants had to learn the correct combinations of numbers and buttons through trial-and-error.

Unbeknownst to participants, two features of the task systematically induced uncertainty. First, for approximately 10% of the trials, they received negative feedback even when the response was correct. Second, after 33–48 trials, the number-button combination changed to a new combination with no prior notice. Each block of 33–48 trials with a single combination was termed an episode. The full task comprised 24 episodes with three unique combinations of numbers and buttons that recurred over time. Owing to the uniqueness of combinations, after a few episodes, it was assumed that the new combinations were extracted from memory rather than learned once again. This feature of the task allowed us to focus on the exploitation-exploration dilemma in the context of alternative strategies’ retrieval.

### Simulation of cathodal HD-tDCS with SimNIBS

**Fig. 1:**
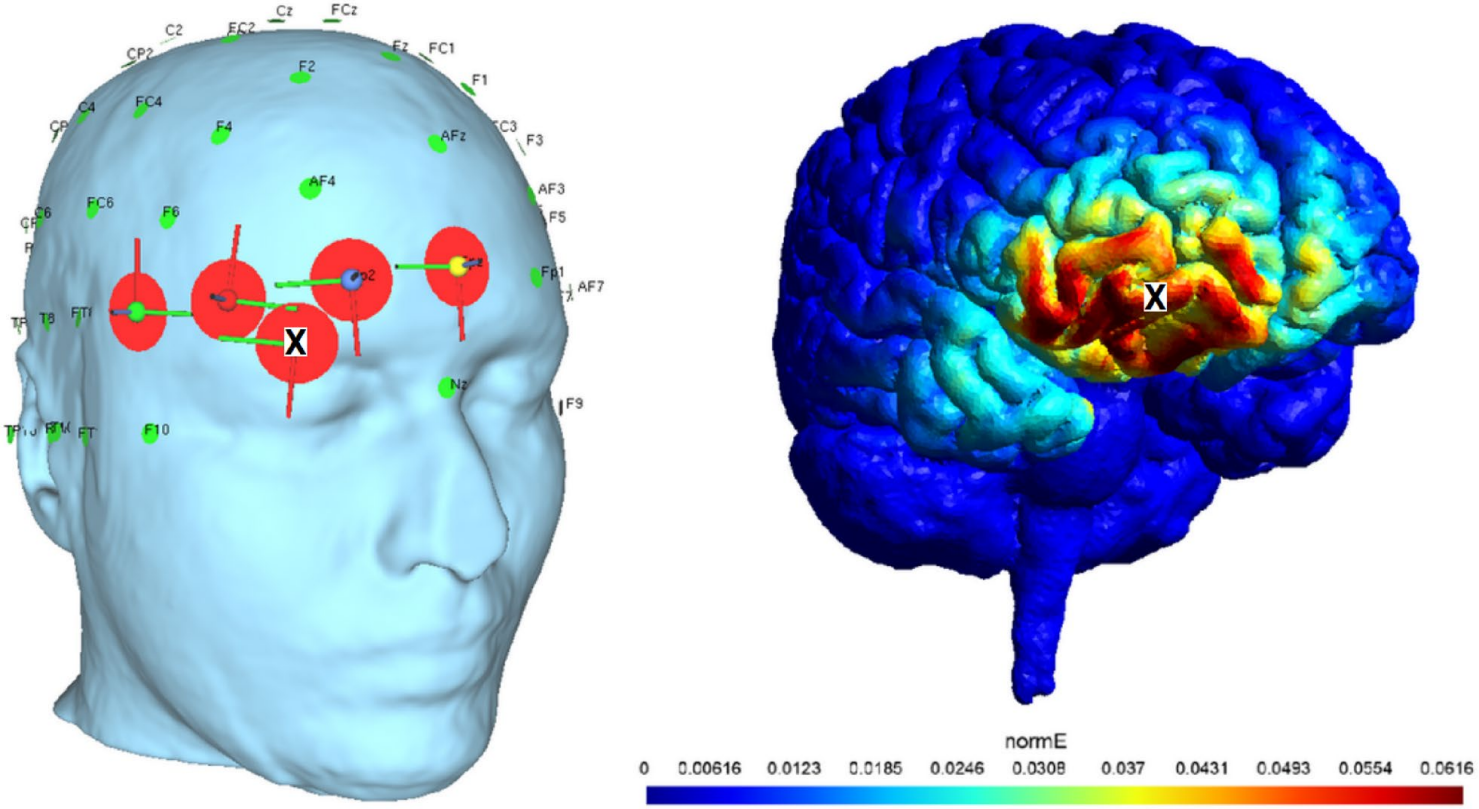
Results of SimNIBS simulation. **Left**: HD-tDCS montage for the rFPC (right frontopolar cortex). Black cross indicates correspondence of cathodal electrode position between both views. **Right**: simulation of the stimulation protocol with SimNIBS. A focused effect of the stimulation is found on the rFPC. The simulation is based on the MRI reconstruction of one of the authors.

The cathodal HD-tDCS protocol was employed to inhibit the activation of the right FPC. This protocol comprised 15 min of stimulation with anodal electrodes over AF8, FPz, F8, and Fp2, with a single cathodal electrode over the right eyebrow (see Fig. 1, left). Prior to data collection, we simulated our protocol with SimNIBS software^10^ and confirmed the focus of the stimulation over the right frontopolar cortex (see Fig. 1, right) with the MNI coordinates from the previous fMRI study^5^. A preliminary test of the protocol revealed no statistically significant difference in the stimulation intensity sensations between cathodal and sham stimulation.

Although prior fMRI findings^5^ suggested bilateral involvement of the frontopolar cortex in strategy monitoring and evaluation of alternatives, the present protocol targeted the right FPC specifically. This decision was informed by recent causal evidence suggesting functional lateralization within the FPC. Notably, in one study^21^ inhibitory rTMS applied unilaterally over the right FPC caused selective disruption of directed exploration, while leaving random exploration unaffected. These findings imply that the right FPC plays a critical and possibly dominant role in monitoring and shifting between alternative strategies under uncertainty. Therefore, targeting the right FPC alone allowed us to causally test its specific contribution while minimizing unintended stimulation of other prefrontal areas.

### No effect of cathodal HD-tDCS on exploratory behavior

**Fig. 2:**
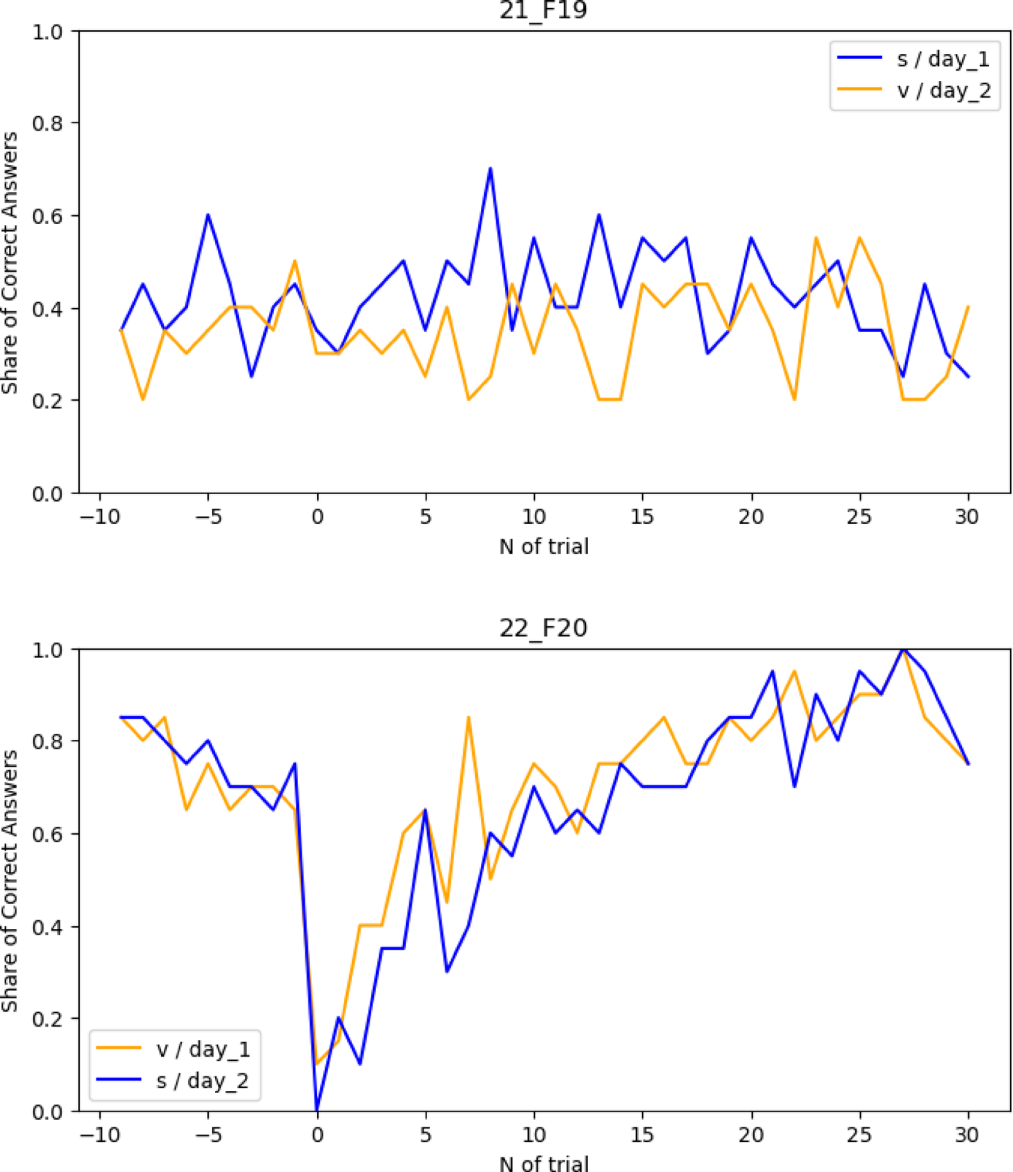
Task performance curves around the combination switch over episodes from 5 to 24 for two participants. The first four episodes were excluded due to the assumption that at least four episodes are required to learn three combinations and start retrieving them from memory rather than relearning them. A trial number of 0 represents a switch to a new episode and a change of the correct combination. This switch results in a sharp drop in performance and elicits an exploration period. “V” in the legend stands for verum stimulation, “S” stands for sham stimulation. **Upper**: Example of the performance curve of the only participant who performed the task completely at random. **Down**: Example of the performance curve of the participant who understood the task.

Task performance curves were first examined around combination switches across episodes 5–24. The initial four episodes were excluded under the assumption that a minimum of four episodes was required to learn three combinations and start retrieving them from the memory rather than relearning them. This resulted in the exclusion of one participant who performed in the task at random (see Fig. 2). The final sample for analysis therefore consisted of forty-two participants.

Second, the PROBE model (described in the previous work^4,5,9^ was fitted to the behavioral data of each participant. This model approximates Dirichlet processes mixtures^3^ and integrates Bayesian techniques with both model-free and model-based reinforcement learning. The model formalizes the concept of task set (i.e., the combination of numbers and buttons that guide the ongoing behavior of the agent) and task set reliability, as well as task selection and creation, action selection and learning, and several other processes. Using genetic algorithms, eight model parameters were estimated separately for each day of each participant by maximizing the likelihood of observed responses.

**Fig. 3:**
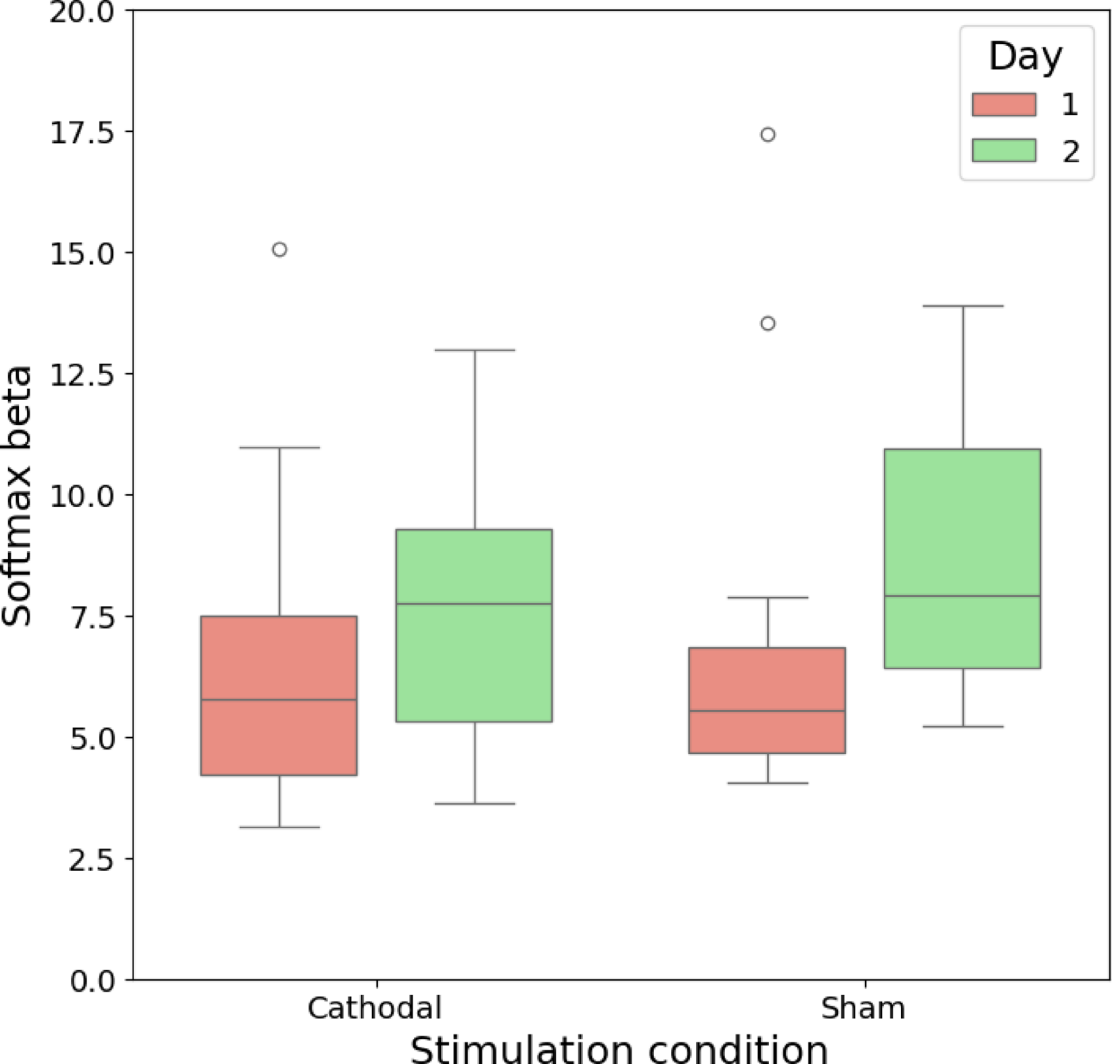
Estimated softmax beta parameters across days and stimulation conditions. No significant differences were observed. Note that for this figure, the Y-axis is cropped to values from 0 to 20 for more intuitive visualization. See Figure S1 for the full plot with outliers included.

Among the fitted parameters, the inverse temperature, or softmax beta, is often assumed to govern the balance between exploration and exploitation^1,11^. As the softmax beta increases, the balance shifts toward exploitatory behavior. Additionally, we assumed that the learning effect for the task structure was also present and could affect the results. Thus, we examined the influence of study day and stimulation condition on softmax beta. Two-way repeated-measures ANOVA did not reveal any significant differences in the estimated softmax beta parameters for different days (F(1, 40) = 2.624, *p* = 0.11) or for either type of stimulation (F(1, 40) = 0.673, *p* = 0.42) (see Fig. 3). Thus, cathodal stimulation of the frontopolar cortex did not significantly alter the balance between exploration and exploitation, as captured by the estimated softmax beta parameters of the PROBE model.

**Fig. 4:**
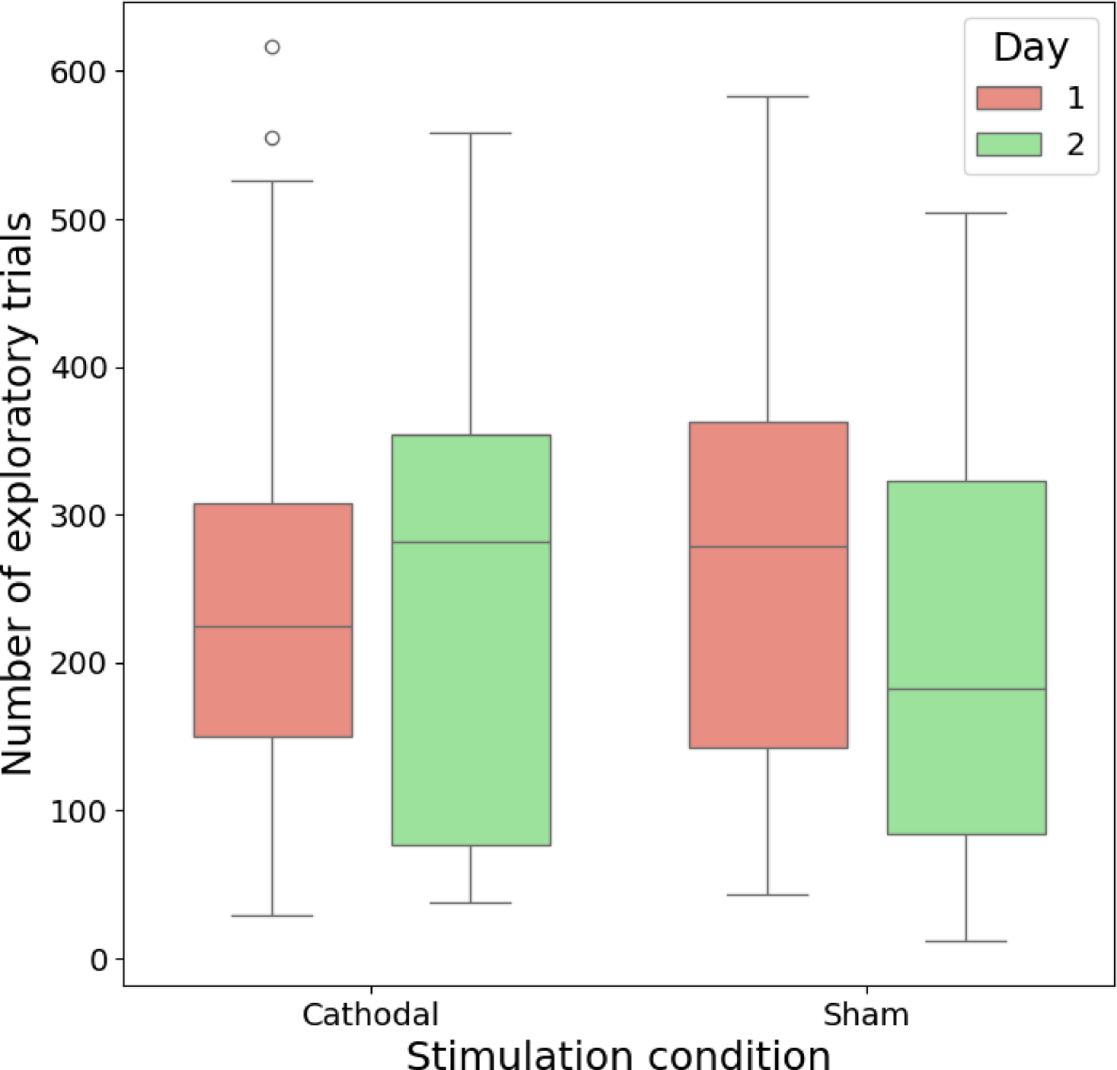
Estimated number of exploratory trials as simulated with the PROBE model. Fitted parameters for the behavioral data were used for the simulations.

As an additional test of our hypothesis, we classified each trial as exploratory or exploitative and compared the number of exploratory trials across days and stimulation conditions. The PROBE model can perform this classification by estimating the reliability of the current task set, which guides ongoing behavior in the simulated behavior. While the task set is reliable (p(task set is reliable) > = 0.5), a trial is classified as exploitatory. When it becomes unreliable (p(task set is reliable) < 0.5), the period of exploration starts and lasts until the new task set becomes reliable. Given that there are only three unique combinations in the experimental paradigm, the end of the exploration period is treated as the retrieval of the counterfactual strategies rather than learning the new strategy. To focus on strategy retrieval, the first four episodes were excluded, as they were assumed not to capture the process of interest. Two-way repeated-measures ANOVA did not reveal any significant differences in the number of exploratory trials for different days (F(1, 40) = 0.881, *p* = 0.35) or for either type of stimulation (F(1, 40) = 0.154, *p* = 0.70) (see Fig. 4; mean numbers of exploratory trials in each condition are presented in the Table S3). Thus, cathodal stimulation of the frontopolar cortex did not shift the balance between exploration and exploitation, as indicated by the total number of exploratory trials simulated by the PROBE model.

### No effect of cathodal HD-tDCS on resting-state fNIRS

To assess the physiological impact of stimulation, resting-state functional near-infrared spectroscopy (fNIRS) was recorded before and after cathodal HD-tDCS. Generally, anodal tDCS is expected to increase neural activity and therefore the oxygenated hemoglobin concentration (HbO) in the resting state, whereas cathodal tDCS is expected to decrease neural activity and HbO^12,13^. Accordingly, fNIRS was expected to confirm the inhibitory effect of tDCS on the rFPC (namely, the right frontopolar cortex) by revealing a decrease in HbO levels after stimulation.

**Fig. 5:**
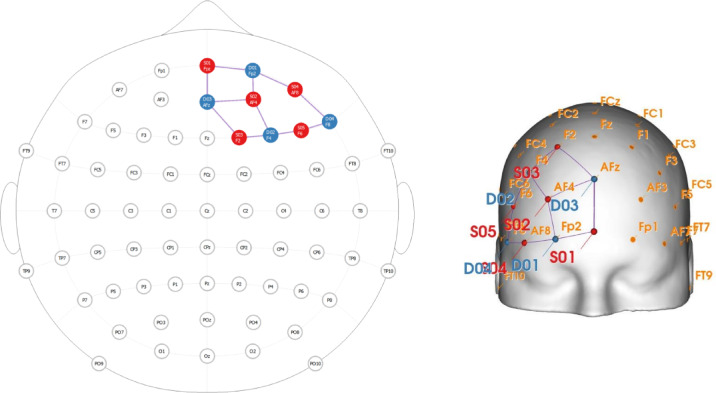
**Left**: fNIRS montage for the right prefrontal cortex. The red dots represent sources of light, whereas the blue dots represent detectors. The channels of interest are those three with Fp2 included. **Right**: 3D model of the montage acquired with NIRSite software. Fp2 is the closest to the stimulated area. In practice, during test tDCS-fNIRS sessions, the position of Fp2 coincided with the position of the cathodal electrode.

The fNIRS montage was designed in line with the HD-tDCS protocol simulation (Fig. 5). Channels with detector D01 over Fp2, the closest site to the stimulation area, were the main channels of interest and were used for further analysis. Because both oxygenated and deoxygenated hemoglobin concentrations (HbO and HbR, respectively) are typically negatively correlated^14^, for further analysis, only channels with HbO were considered. For each participant, three 5-minute resting-state recordings were obtained per condition (cathodal and sham): one before stimulation, one immediately after stimulation, and one following task completion, resulting in six recordings per participant. For each recording, the signals from channels S01_D01, S02_D02, and S04_D01 were averaged.

**Fig. 6:**
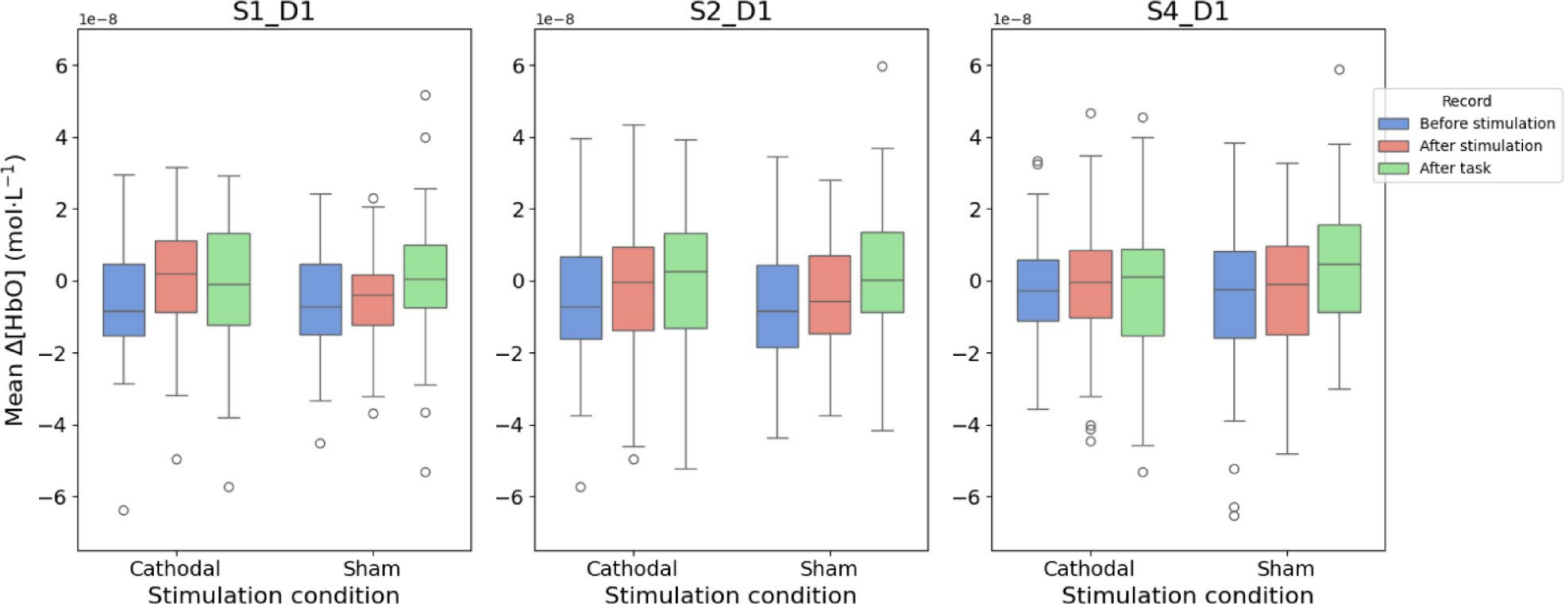
Averaged resting-state fNIRS signals for each channel of interest. After Beer–Lambert conversion, signals represented relative changes in concentrations (mol/liter) of oxyhemoglobin (HbO). The channels of interest are those with a detector set over Fp2, the closest point overlapping with the stimulation area. No significant difference was revealed between the type of stimulation and the time of the recording.

Preprocessing included quality control, motion correction, and conversion to optical density and relative HbO concentrations. To evaluate stimulation effects, a two-way MANOVA was performed with average HbO signals as dependent variables, and stimulation type (sham vs. cathodal) and recording time (pre-stimulation, post-stimulation, post-task) as independent variables. The test revealed no effect of recording time (Roy’s greatest root = 0.01, F(3, 251) = 1.24, *p* = 0.30), stimulation type (Roy’s greatest root = 0.01, F(3, 250) = 0.81, *p* = 0.49), or interaction of these factors (Roy’s greatest root = 0.01, F(3, 251) = 1.17, *p* = 0.32). Group-level results are shown in Fig. 6. In summary, cathodal HD-tDCS did not induce detectable changes in resting-state HbO concentrations in the rFPC.

### Learning effect on the total number of correct responses

**Fig. 7:**
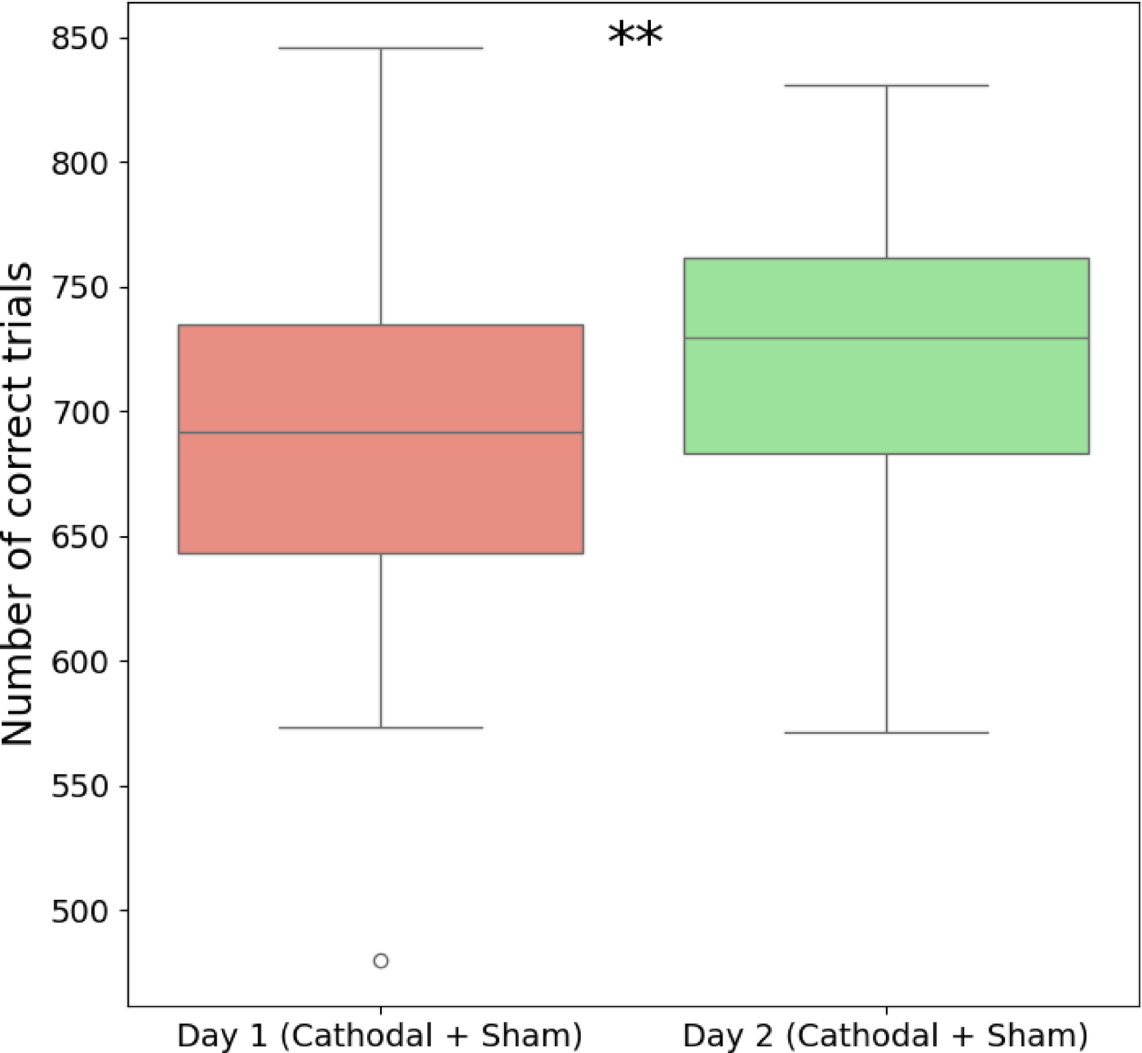
The number of correct trials was significantly greater on the second day of the experiment, indicating a learning effect on the task. Note that only the structure of the task was similar: the presented numbers and combinations were different for each day. **: p-value < 0.01.

Because participants had to perform the task twice, we expected that the learning effect might confound the results. Although different sets of numbers and combinations were used each day, a two-way repeated-measures ANOVA revealed a significant increase in the number of correct trials on the second day (F(1, 40) = 10.791, *p* = 0.002; Cohen’s d = 0.55) and no effect of stimulation (F(1, 40) = 0.27, *p* = 0.61) (see Fig. 7; mean numbers of correct trials in each condition are presented in the Table S2).

**Fig. 8:**
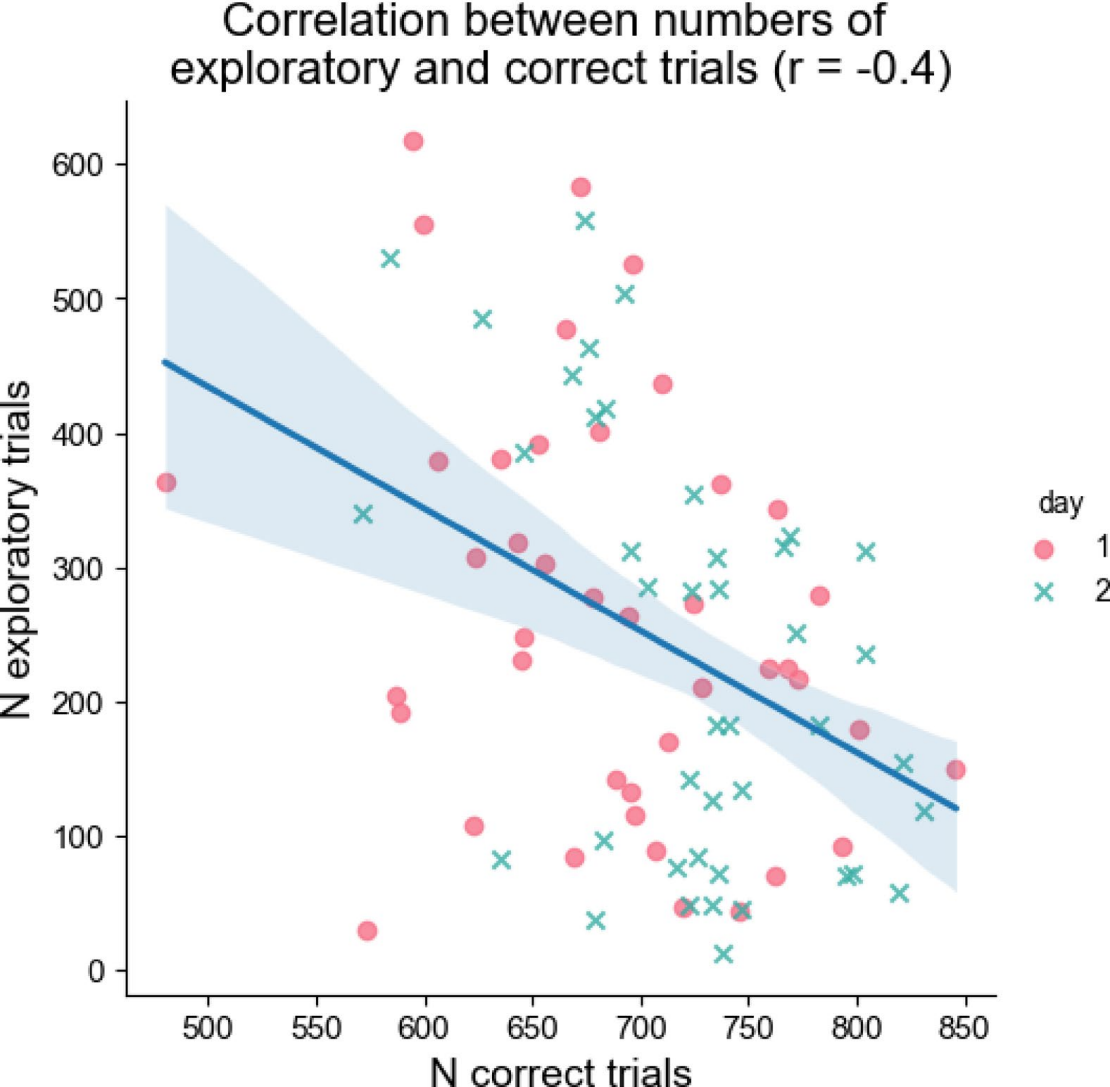
Relationship between the number of exploratory and correct trials. Each dot represents the data either for day one or for day two, regardless of stimulation type.

To further explore performance, a bootstrapped Pearson correlation (10,000 resamples) was conducted between the number of exploratory trials and the number of correct trials. The mean correlation coefficient was *r*=−0.40, with a 95% confidence interval ranging from − 0.54 to −0.21, indicating a moderate to strong negative association (Fig. 8; full distribution of bootstrapped Pearson correlations is presented on Figure S2).

### The models perform better than humans do

**Fig. 9:**
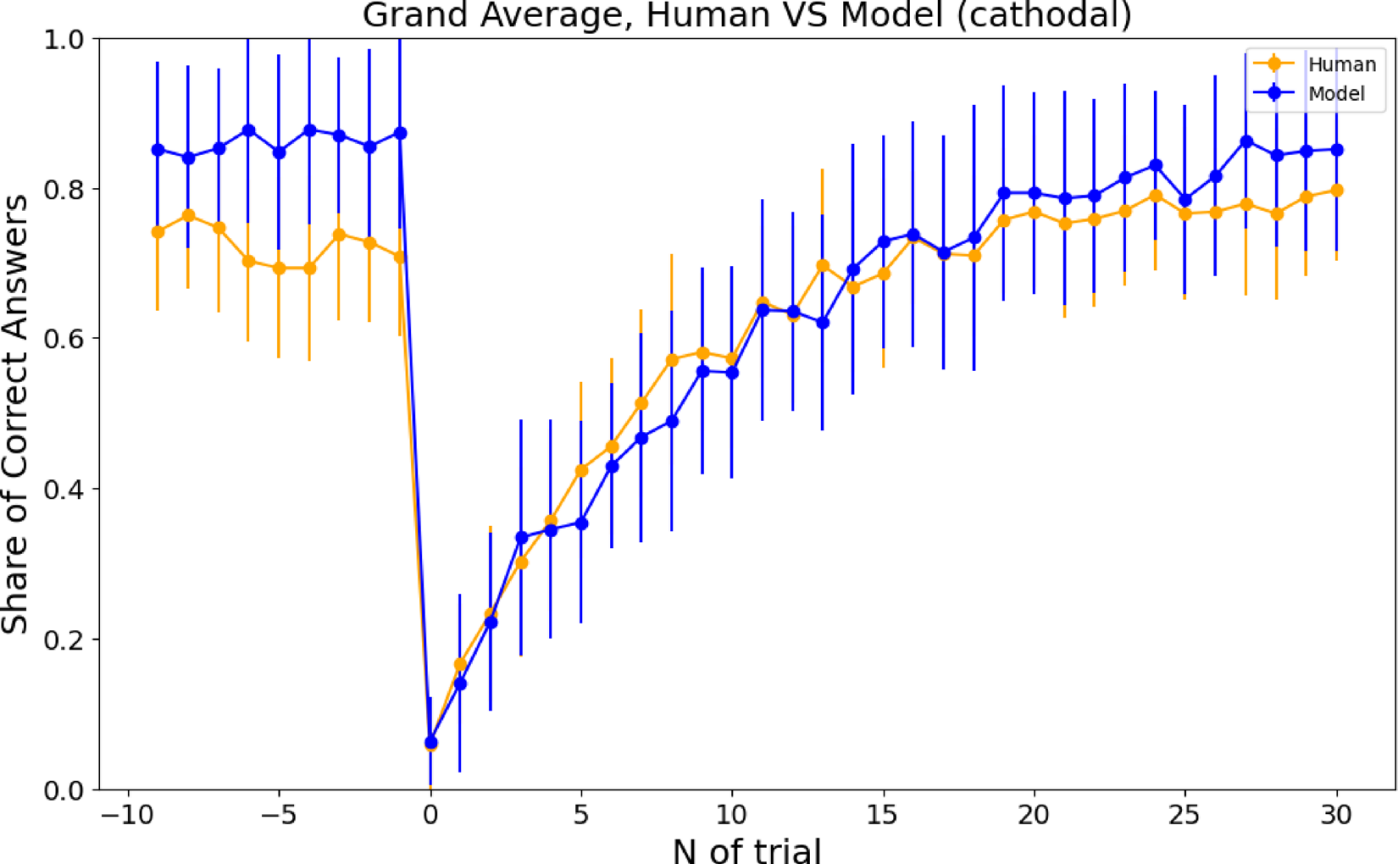
Average performance around the combination switch for behavioral data and model simulations with the corresponding fitted parameters. Only behavioral data and corresponding fitted parameters from the cathodal condition are shown. The model tends to perform better at the end of the exploitation period, immediately before the combinations switch. Otherwise, the behavior is similar. Each dot represents the mean and standard deviation of the share of correct responses averaged across all participants.

**Fig. 10:**
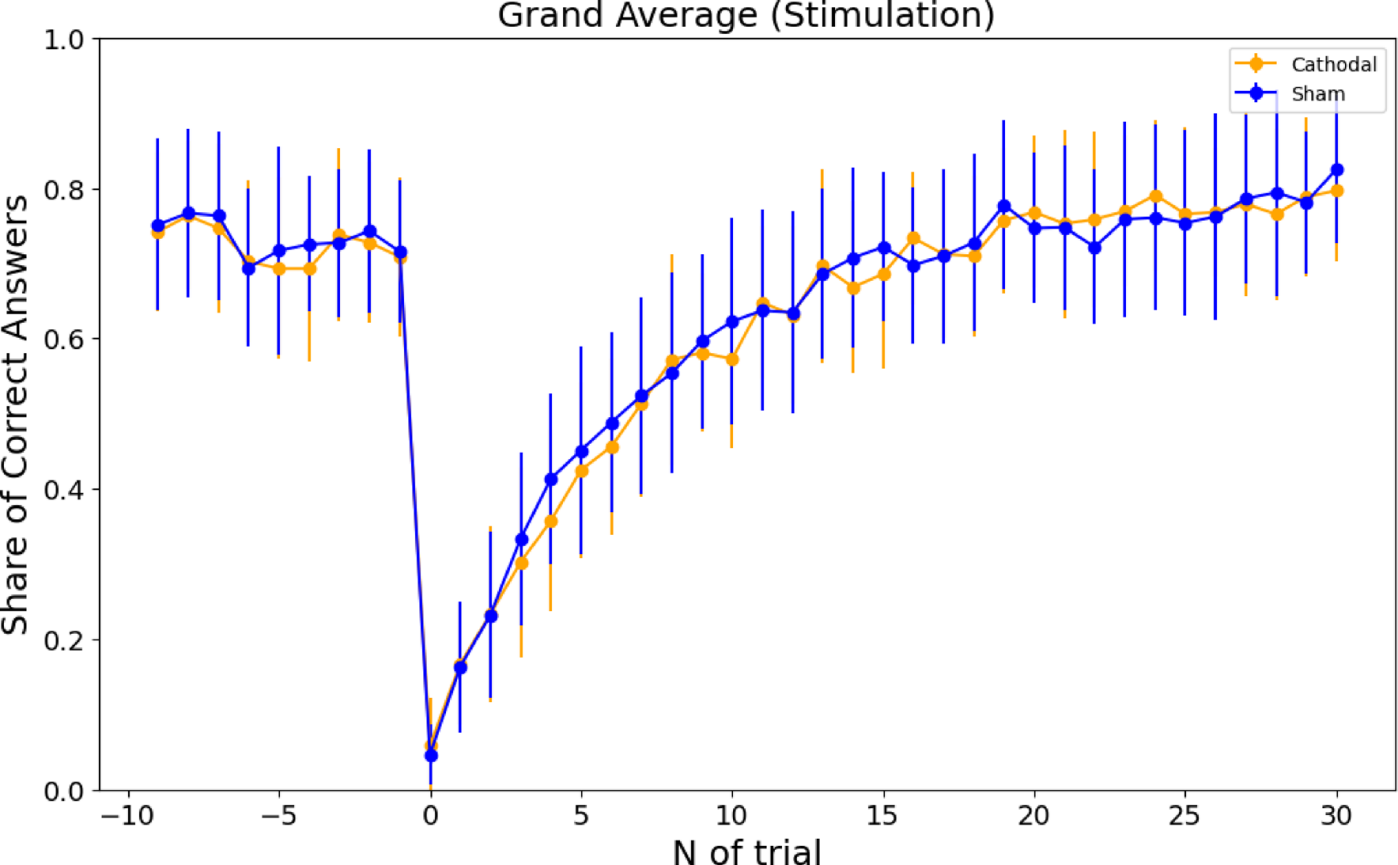
Average performance around the combination switch for behavioral data (cathodal versus sham conditions). The performance does not differ between the conditions. Each dot represents the mean and standard deviation of the share of correct responses averaged across all participants.

The PROBE model is a complex model that requires the estimation of at least eight parameters^4,5,9^ (see Tables S1 and S4). Using parameters fitted to the behavioral data, we simulated model performance and compared average accuracy around combination switches between simulated agents and human participants. Simulations showed that the outperformed humans immediately before the combination switch, at the end of the exploitation phase. As in humans, performance dropped sharply after the switch. Figure 9 illustrates both model and human behavior after cathodal stimulation (see Figures S3-S5 for similar plots in other conditions). Human performance in the same period did not differ across stimulation conditions (see Fig. 10 for human performance after sham versus cathodal stimulation; see Figures S6-S8 for similar plots in other conditions).

### Parameter recovery with genetic algorithms

**Fig. 11:**
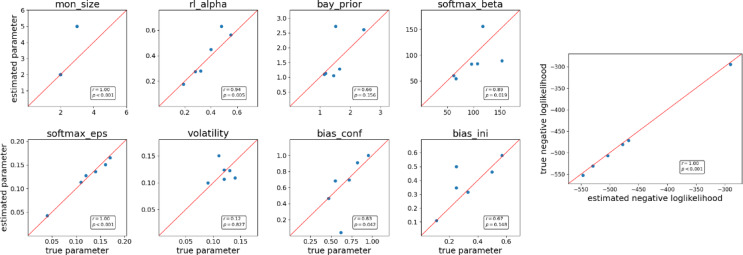
The genetic algorithm finds the sets of parameters that explain the generated data slightly better based on the negative loglikelihood. **Left**: True and best recovered parameters with genetic algorithms (based on the set of parameters in Table S1). Mon_size – Monitoring buffer capacity, rl_alpha – learning rate in reinforcement learning (RL), bay_prior – Bayesian prior, softmax_beta – inverse temperature in RL, softmax_eps - ‘lapse’ rate of softmax (i.e., noise), volatility – perceived volatility, bias_conf – confirmation bias, bias_ini - recollection entropy, LL – loglikelihood of choice probabilities in simulated data. **Right**: Negative loglikelihood of the true and best models.

To validate genetic algorithms as a procedure for parameter estimation, we conducted a parameter recovery study on the six sets of parameters reported in the previous study by Domenech and colleagues (2020)^9^ (Table S1). Because the reported range of the confirmation bias parameter differed from the provided MATLAB code, these parameter values were instead taken from Donoso et al. (2014)^5^. First, using each set of parameters, we simulated behavior on the same set of trials and computed negative loglikelihood as a measure of model fit. Next, with genetic algorithms, we re-estimated the parameters of the PROBE model. Although not all recovered parameters exactly matched the true values, the recovered sets yielded slightly improved negative log-likelihoods (Fig. 11). This means that the genetic algorithms resulted in slightly better parameter estimation for the generated data and validated our procedure of model fitting.

## Discussion

The aim of this study was to employ the PROBE model and its corresponding task to causally test the role of the human frontopolar cortex (FPC) in managing counterfactual task sets during decision-making in uncertain and changing environments. Based on previous findings, we hypothesized that inhibition of the right FPC with cathodal HD-tDCS would result in more exploratory behavior than for sham stimulation. We tested this hypothesis in two ways. First, by fitting the PROBE model parameters to behavioral data and comparing the inverse temperature parameter across conditions. Second, by classifying trials as exploratory or exploitatory based on model-derived estimates of task set reliability and comparing numbers of exploratory trial across conditions. Neither approach revealed any significant differences between stimulation conditions and, thus, did not confirm our hypothesis.

Prior fMRI findings consistently revealed that the frontopolar cortex was activated during exploratory decisions both in the same task as the PROBE model^5^ and in other tasks, such as the armed bandit task^15,16^ and the temporal utility integration task^17^, but not in the dynamic version of the “observe or bet” task^18^. In another study, it was specifically associated with the switch to the alternative strategy in the armed bandit task^19^. Moreover, causal confirmation of the role of the frontopolar cortex in exploration was previously reported^20,21^. Notably, inhibitory rTMS over the right FPC selectively disrupted directed exploration, while leaving random exploration intact^21^, and cathodal tDCS in a bandit task reduced exploration, whereas anodal stimulation increased it^20^. These findings contradict both the predictions of the PROBE model and our hypothesis.

Several factors may account for these discrepancies between our results and literature. First, although SimNIBS simulations of the tDCS protocol showed the effect of stimulation on the rFPC, the high-definition protocol may have been too weak when applied in practice. This interpretation is supported by the absence of stimulation effects in our resting-state fNIRS data. This problem could be solved either by applying a conventional tDCS protocol, or by applying continuous theta-burst stimulation, which was also claimed to inhibit activation of the targeted area for up to 50 minutes^22^.

Second, the within-subject design of the study implied that participants had to perform the task twice, with a one-week break, potentially introducing learning effect. Despite using different button-number combinations, on average, there were more correct responses on the second day than on the first day. Thus, knowing the task structure made learning easier on the second day. Furthermore, the numbers of correct trials and exploratory trials were not independent but negatively correlated (Fig. 8 and Figure S2), with a correlation coefficient of approximately − 0.40.

Given this fact, we assume that the learning effect might also change tendencies in exploratory behavior. A between-subject design could mitigate this limitation, although it would require careful control of potentially confounding variables, such as IQ, socioeconomic status, gender, age, and other possible intervening variables.

The last reason for these contradictory results might be the complexity of the PROBE model. Our parameter recovery analyses showed that while some parameters (e.g., learning rate, noise) were recovered reliably, others (e.g., inverse temperature) deviated substantially from true values, likely due to model complexity and interdependencies among parameters. We are convinced that this difference in parameters could arise from the complexity of the PROBE model, which includes eight free parameters.

Mostly, the estimated parameters in our study were comparable to the previously reported^5,9^ (see estimated parameters for this study in Table S4). A few exceptions were seemingly higher perceived volatility and monitoring buffer capacity and lower recollection entropy. However, this comparison has rather limited and exploratory value: there were only means and standard errors of the estimated parameters reported by Donoso and colleagues (2014)^5^, whereas Domenech and colleagues (2020)^9^ provided estimations for each of their six participants who were recruited from the clinical sample, not the general sample, as in this study.

Despite these limitations, our study extends prior work in several ways. First, we implemented a high-definition transcranial direct current stimulation (HD-tDCS) protocol informed by preliminary simulations. This approach presumably offers greater spatial precision than traditional tDCS does, potentially leading to more targeted modulation of frontopolar cortex activity, although our stimulation protocol was not found to be effective. Second, we employed the PROBE model, which not only captures behavioral choices but also classifies each trial as exploratory or exploitatory, providing a richer characterization of behavior. Third, we utilized functional near-infrared spectroscopy (fNIRS) to monitor changes in physiological responses for stimulation.

In conclusion, although our findings did not support the hypothesized causal role of the rFPC in managing alternative strategies, they underscore important methodological considerations for future research. Our study highlights the need for further refinement of HD-tDCS protocols targeting the frontopolar cortex for the modulation of exploration behavior. Employing alternative neurostimulation techniques, such as rTMS, may prove more effectice for future research. Moreover, further development of the PROBE model and, broader, prefrontal executive function theory might also benefit from our results by providing more data on how real-world decision-making behavior aligns with the PROBE model predictions.

## Methods

### Participants

Forty-three participants participated in this study (19 males, mean ± std age 21.1 ± 2.4 years, range = 18–29 years). Only right-handed and healthy participants who were not taking any medication for neurological or psychiatric illnesses or other psychotropic drugs were included. Having any metal in the head was another criterion for exclusion. Smokers were not excluded from the study. Each participant had to come twice with a one-week break to receive either sham or cathodal stimulation. Half of the participants received sham stimulation on the first day, and another half on the second day, which allowed us to distinguish between the learning effect on the task and the stimulation effect. All participants completed a modified screening questionnaire^23^ before the study. Additionally, all participants were paid after the experiment depending on their performance during the task. Note that a between-subject design would require strict control of additional measurements between groups (e.g., IQ, impulsivity, risk-taking behavior), whereas the within-subject design of this study allows us to diminish the effects of these differences.

All participants read and signed the written informed consent form prior to the experiment. All procedures were approved by the research ethics committee of the HSE university. All methods were carried out in accordance with relevant guidelines and regulations.

### Experimental paradigm

The task imitates an uncertain and changing environment that systematically induces resolving exploitation-exploration dilemmas in each trial. During the task, an Arabic number (1, 3, or 5 for one day; 2, 4, or 6 for another day) is presented on the screen in four boxes simultaneously. The participant must respond to each number by pressing one of four response buttons. After the button is pressed, the participant is provided with feedback—either positive or negative. Positive feedback is the green-colored number in the box displayed on the screen and represents the chosen button. Negative feedback is a red cross in the box representing the chosen button. If no button was pressed, red lines appeared in the boxes. Each correct response increased the reward for the participant after the experiment. Uncertainty in the task is elicited in two ways: first, feedback is not entirely reliable: in 10% of trials, even if the correct response was given, one could receive negative feedback. Second, systematically, after a variable number of trials, the correct combination will change with no prior notice. The goal of the task is to determine the correct combinations of presented numbers and response buttons. Thus, the exploration-exploitation dilemma is induced by the decision to stay with the current combination or to try another combination. One session is divided into 6 blocks of 24 episodes in total (4 episodes per block). Each episode included 33–48 trials. For each session, three unique combinations recurrently appeared across all 24 episodes in a randomized order. The code of the experimental paradigm was provided by the authors of the original study (Donoso et al., 2014) and was written with Psychtoolbox for MATLAB.

### tDCS

Cathodal tDCS is generally assumed to inhibit the activation of the neuronal population under the electrodes^24,25^. For this study, a Starstim stimulator (https://www.neuroelectrics.com*)* with 8 channels was used. To achieve focal inhibition of the right FPC, the high-definition tDCS (HD-tDCS) protocol was applied with 4 anodal sponge electrodes over AF8, FPz, F8, and Fp2 (in accordance with the 10–10 international system) and one cathodal sponge electrode over the right eyebrow (a current intensity of 1.5 mA for the cathodal electrode, 25% stimulation return for each anodal electrode, and 8 cm^2^ for each electrode) (see Fig. 1, left). Stimulation lasted for 15 min, with 10 s for ramp-up, 10 s for ramp-down and 30 s for the sham ramp.

While HD-tDCS designs often use one anode and multiple cathodes to enhance neural activity, our reversed polarity montage aligns with the objective of inhibiting a targeted region. The choice of this configuration was further supported by simulations conducted in SimNIBS (https://github.com/simnibs/simnibs*)* with structural MRI of one of the authors of this study (see Fig. 1). This simulation confirmed a focused current distribution over the rFPC. Such polarity-specific montages have been used successfully in prior neuromodulation studies to investigate causal mechanisms of exploration behavior^20^.

Notably, the conventional 4 × 1 ring configuration was not optimal for our study due to limitations in placing electrodes precisely over the FPC without overlapping with other frontal areas (e.g., DLPFC). Furthermore, in case of the ring configuration, cathodal electrode should have been placed right above the eyebrow, making it impossible to place one of the anodal electrodes below. Therefore, we had to look for and to validate linear configuration of the electrodes through SimNIBS simulations to achieve optimal focality of stimulation.

### fNIRS

For this study, the NIRScout Extended system and NIRX software at the Higher School of Economics, Moscow, were used (https://www.hse.ru/cdm-centre/fnirs*).* The montage was developed in accordance with the tDCS protocol simulation (see Fig. 2 for the fNIRS protocol). The sampling rate was 12.5 Hz, and the wavelengths used were 785 nm and 830 nm, with 11 channels in total. The 3D model of the fNIRS montage with the NIRSite (https://nirx.net/nirsite*)* and preliminary montage test demonstrated that the Fp2 position for the detector optode would be the closest position to the area of the stimulation. Thus, the pairs of source and detector with Fp2 included were the main channels of interest. For the analysis of the effect, 5-minute resting states were recorded three times: before and immediately after stimulation and immediately after paradigm completion.

### Procedure

The experiment was conducted at the fNIRS laboratory at the Higher School of Economics, Moscow. Participants came to the laboratory and were asked to provide informed consent before the experiment and to complete the screening questionnaire. Then, the 5-minute resting state was recorded with fNIRS. Before each recording, the optodes were calibrated in the dark room. During the recording, the participants were required to sit with their eyes open and relax in the experimental room. After the first recording, 15 min of tDCS (sham or verum) stimulation was applied. Immediately after the stimulation, another 5-minute resting state was recorded before the task. Then, the participants read the instructions for the main task and performed a training session, which was a simplified two-minute version of the original task. The training session lasted for 3 min, and its goal was to find a combination of two buttons and two figures (either circle or rectangle). After the training session, the participants had to perform one experimental session consisting of 24 episodes, ranging from 33 to 48 trials per episode and including one out of three unique combinations per episode. After the completion of the experimental paradigm, a 5-minute resting state was recorded with fNIRS for the last time. The overall duration of the experiment was approximately 1 h 50 min, with tDCS and fNIRS preparation included. The procedures for the first and second days differed only in the presence of short training sessions (presented only on the first day), kind of applied stimulation (sham or verum), and numbers used in the task ({1, 3, 5} or {2, 4, 6}).

### fNIRS preprocessing and analysis

The raw signal of fNIRS-measured light intensities was converted to optical density. The quality of the signal was then checked with the Scalp Coupling Index (SCI). The SCI ranges from 0 to 1, and the higher this value is, the better the quality is. The authors of the SCI in the original study^26^ used 0.75 as the threshold SCI value for a channel to be good, even though it may differ across studies. In this research, 0.75 is also chosen as the threshold SCI value. Thus, four channels (namely, S3_D3, S3_D2, S2_D3, and S2_D2) were completely removed from further analysis because of the low quality of more than 5 recordings in total. None of them included a detector on Fp2, which is the closest to the area of stimulation. For 7 other channels, temporal derivative distribution repair was applied to optical densities, which is a method of motion correction for fNIRS^27^. Then, the optical densities were converted to oxyhemoglobin and deoxyhemoglobin concentration changes (in mol/liter), ∆HbO, and ∆HbR, respectively. A fourth-order Butterworth band-pass filter was used for the 0.01–0.1 Hz frequency band. Note that to reduce the influence of cardiac signal, respiration, and Mayer waves, studies that focus on fNIRS and resting states usually include low-pass filter with frequencies below 0.1 Hz^28–30^ or band-pass filter with the lower cutoff frequency set to 0.01 Hz^31–33^. For preprocessing the fNIRS data, the MNE-Python library (https://mne.tools/stable*)* was used.

For the analysis of the HD-tDCS effect, we considered only three channels with the main detector D01 over Fp2, which is the area where changes in the HbO level were expected: S01_D01, S02_D01, and S04_D01 (see Fig. 2). The average signal for each 5-minute recording was computed after preprocessing for each channel. Thus, there were six average values per channel: three for the sham condition and three for the cathodal condition. Among three recordings per condition, one was recorded before the stimulation, one was recorded immediately after the stimulation, and one was recorded after the end of the task. To detect possible differences in the average signals, multivariate analysis of variance (MANOVA) was applied. The dependent variables were the average values of the recordings for each of the three channels with the detector over Fp2, resulting in three variables per recording for each participant. The independent variables were the *type of stimulation* (sham or cathodal) and the *time of the recording* (before the stimulation, after the stimulation, or after the experiment). If significant differences were detected, pairwise Tukey’s ‘Honest Significant Difference’ method would be applied for post hoc analysis. Implementations of MANOVA and Tukey’s test from statsmodels (https://www.statsmodels.org/stable/*)* package in Python were used^34^. The approach to the analysis was partly inspired by another paper^35^.

### Computational modeling

The PROBE model used in this study requires the fitting of eight free parameters: monitoring buffer capacity (N), learning rate in reinforcement learning (α), Bayesian prior, or weight of flat priors for a newly created task set (π), inverse temperature in reinforcement learning (β), ‘lapse’ rate of softmax (i.e., noise, ε), perceived volatility (τ), confirmation bias for a newly created task set (θ), and recollection entropy (η). The PROBE model computationally describes how the human prefrontal cortex guides adaptive behavior in uncertain, changing, and open-ended environments by approximating optimal adaptive processes (i.e., the Dirichlet processes mixture). This model operates with *task sets* – active representations of behavioral strategies stored in long-term memory. The PROBE model mathematically implements task set creation and selection, action selection, and strategy learning. For a detailed description of the PROBE model, refer to the previous works^4,5,9^. The MATLAB code of the PROBE model was kindly provided by the authors of the original papers.

The central concept of our research is *task set reliability*. For each trial, the PROBE model computes the reliability of the task set, which guides ongoing behavior and infers the reliabilities of N alternative strategies, where N is the monitoring buffer capacity. While the task set is reliable *(p(task set is reliable) > = 0.5)*, the agent stays in exploitation mode. When the task set becomes unreliable (*p(task set is reliable*) *< 0.5)*, the agent switches to exploration mode and continues exploring until one of the monitored task sets becomes reliable. This feature allowed us to classify each trial as either exploratory or exploitatory.

Since we were interested in the retrieval of alternative strategies rather than in creating new reliable strategies, we removed the first four episodes from the main analysis.

### Parameter fitting procedure

We limited the inverse temperature (β) range from 0 to 200, the Bayesian prior (π) range from 1 to 3, the ‘lapse’ rate of softmax (ε) range from 0 to 0.5, and the monitoring buffer parameter with integer numbers ranging from 1 to 6. All other parameters ranged from 0 to 1.

Given these strict limitations on the parameter range and, consequently, the limited parameter space, we decided to use genetic algorithms for parameter fitting. Previously, genetic algorithms were shown to be plausible for point estimation of the best parameters of complex models^36^. We implemented the genetic algorithms in MATLAB R2023b (https://ch.mathworks.com/help/gads/ga.html*)* with the population size parameter set to 50 000 and all other parameters set to defaults. The PROBE model estimates probabilities of choices in each trial, so the optimization goal was to maximize the loglikelihood of participants’ responses in the task. For each participant, genetic algorithms were run six times, once per fixed value of monitoring buffer from 1 to 6.

To check whether this procedure was adequate for this model, we conducted parameter recovery analysis for six sets of parameters on the basis of the parameters reported by Domenech and colleagues (2020)^9^. The range of confirmation bias differed in the provided code of the model and in (Domenech et al., 2020)^9^, so we used plausible values from the confirmation bias range reported in (Donoso et al., 2014)^5^. We computed the loglikelihood of the simulated choices, recovered the parameters via genetic algorithms, and compared the loglikelihoods for the same choice data. Overall, the loglikelihoods for the estimated parameters were slightly higher than those for the ground truth, and most of the parameters were close to the ground truth. See Table S1 for the exact parameters and loglikelihoods.

### Statistical analysis

Bootstrapped Pearson correlation implementation from the SciPy^37^ library for Python was used with 10 000 draws. For repeated-measures ANOVA analysis *aov*, methods from the *ez* library for R language were used. For data visualization, seaborn^38^ and matplotlb^39^ were used.

## Supplementary Information

Below is the link to the electronic supplementary material.

Supplementary Material 1

## Data Availability

The data collected during this study, as well as the scripts used for modeling and analysis, are available here: https://osf.io/fth7a.
