# Correction: No effect of preliminarily simulated cathodal HD-tDCS on the frontopolar cortex in the exploration–exploitation task

**DOI:** 10.1038/s41598-025-32681-9

**Published:** 2025-12-18

**Authors:** Viktor Timokhov, Andrey Timashkov, Oksana Zinchenko

**Affiliations:** 1 Zurich Center for Neuroeconomics, Department of Economics, University of Zurich, Zürich, Switzerland; 2 Centre for Cognition and Decision-Making, Institute for Cognitive Neuroscience, National Research University Higher School of Economics, Moscow, Russia

Correction to: *Scientific Reports* 10.1038/s41598-025-22016-z, published online 31 October 2025

The original version of this Article contained an error in the names of Viktor Timokhov, Andrey Timashkov and Oksana Zinchenko, which were incorrectly given as Timokhov Viktor, Timashkov Andrey and Zinchenko Oksana.

The original Article has been corrected.

