# Supplementary material for: No effect of preliminarily simulated cathodal HD-tDCS on the frontopolar cortex in the exploration-exploitation task
Source: Sci Rep. 2025 Oct 31;15:38153. doi: 10.1038/s41598-025-22016-z (PMC12578884; doi:10.1038/s41598-025-22016-z)
Supplement: Supplementary file 1 — Supplementary Material 1 [file 41598_2025_22016_MOESM1_ESM.docx]

# Supplementary materials


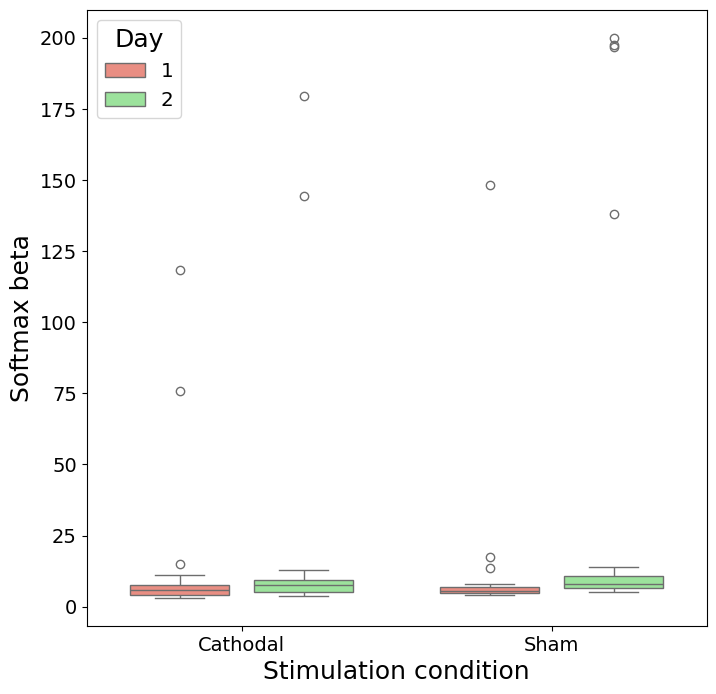


*Figure S1.* Estimated softmax beta parameters across days and stimulation conditions for all participants. For some of them, the estimated softmax beta parameters ranged from 75-180. For more intuitive visualization, Figure 2 in the main text does not include extreme values.


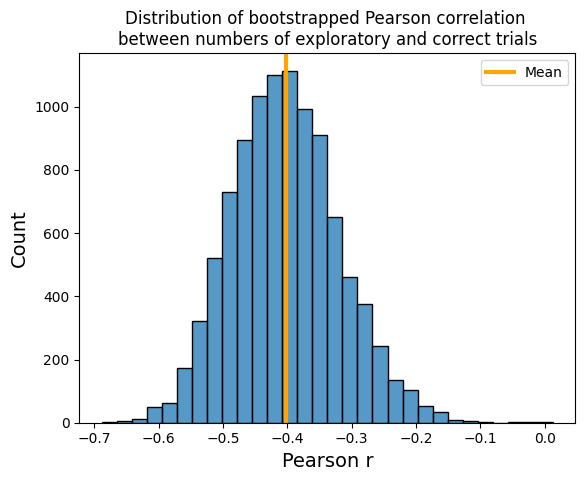


*Figure S2.* Distribution of bootstrapped Pearson correlations between the numbers of exploratory and correct trials. This distribution was acquired with 10 000 draws.


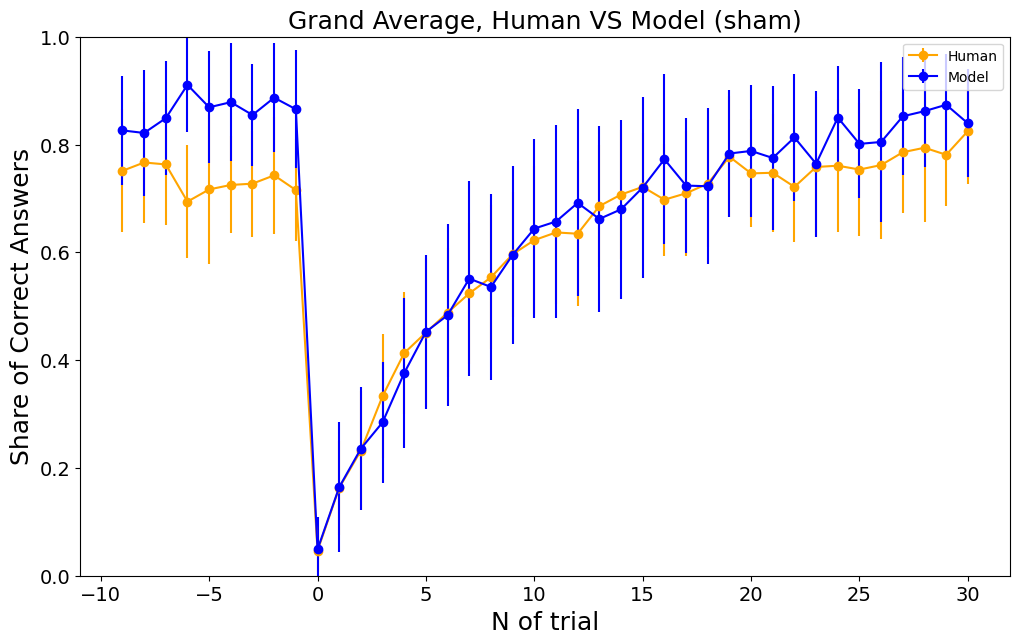


*Figure S3.* Average performance around the combination switch for behavioral data and model simulations with the corresponding fitted parameters. Only behavioral data and corresponding fitted parameters from the sham condition are shown. The model tends to perform better at the end of the exploitation period, immediately before the combinations switch. Otherwise, the behavior is similar. Each dot represents the mean and standard deviation of the share of correct answers averaged across all participants.


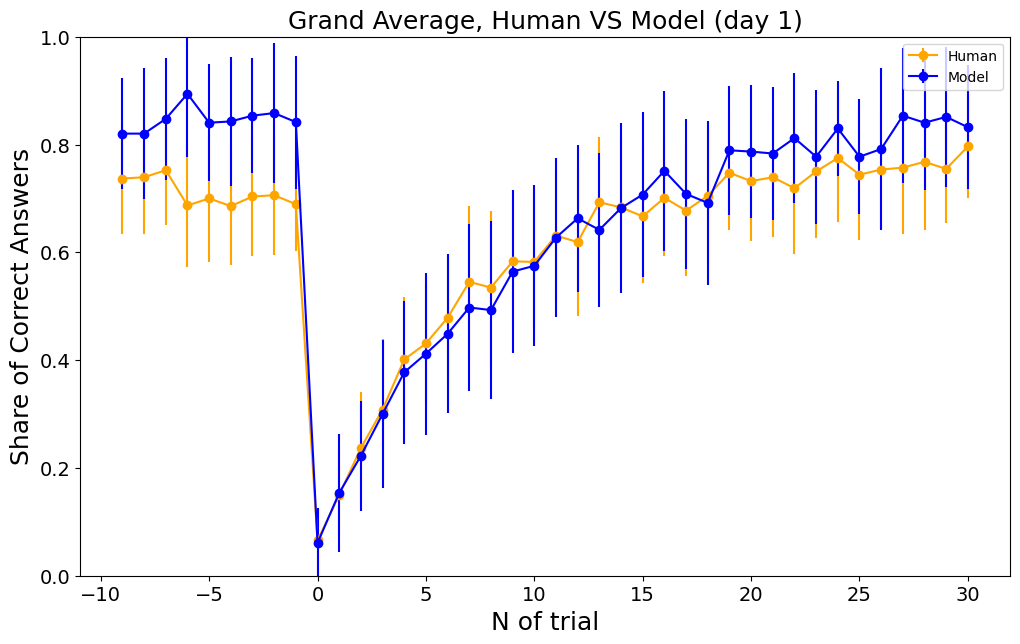


*Figure S4.* Average performance around the combination switch for behavioral data and model simulations with the corresponding fitted parameters. Only the behavioral data and corresponding fitted parameters from day one are shown. The model tends to perform better at the end of the exploitation period, immediately before the combinations switch. Otherwise, the behavior is similar. Each dot represents the mean and standard deviation of the share of correct answers averaged across all participants.


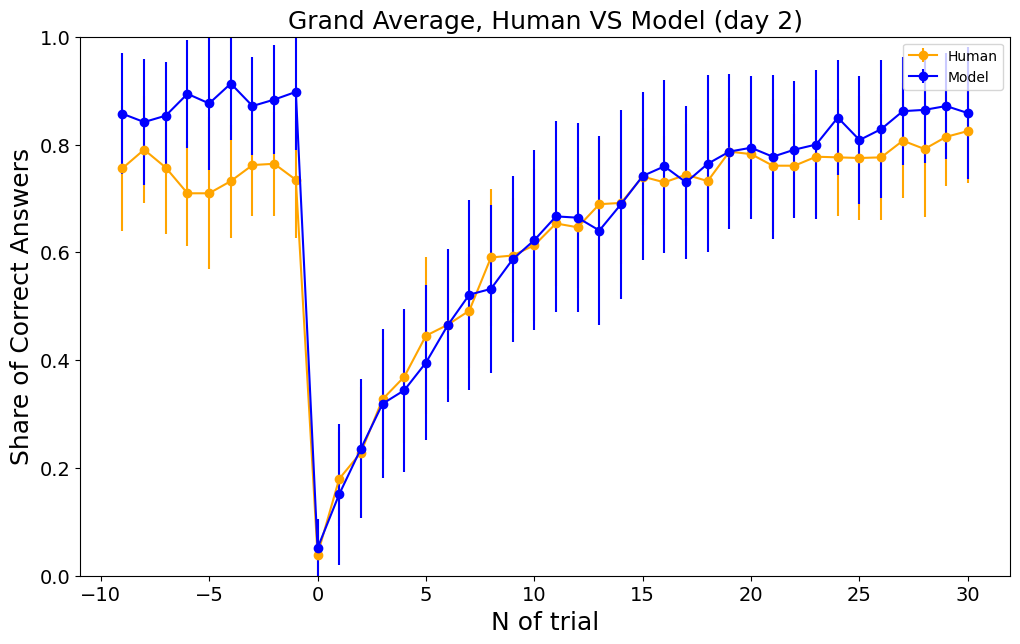


*Figure S5.* Average performance around the combination switch for behavioral data and model simulations with the corresponding fitted parameters. Only the behavioral data and corresponding fitted parameters from day two are shown. The model tends to perform better at the end of the exploitation period, immediately before the combinations switch. Otherwise, the behavior is similar. Each dot represents the mean and standard deviation of the share of correct answers averaged across all participants.


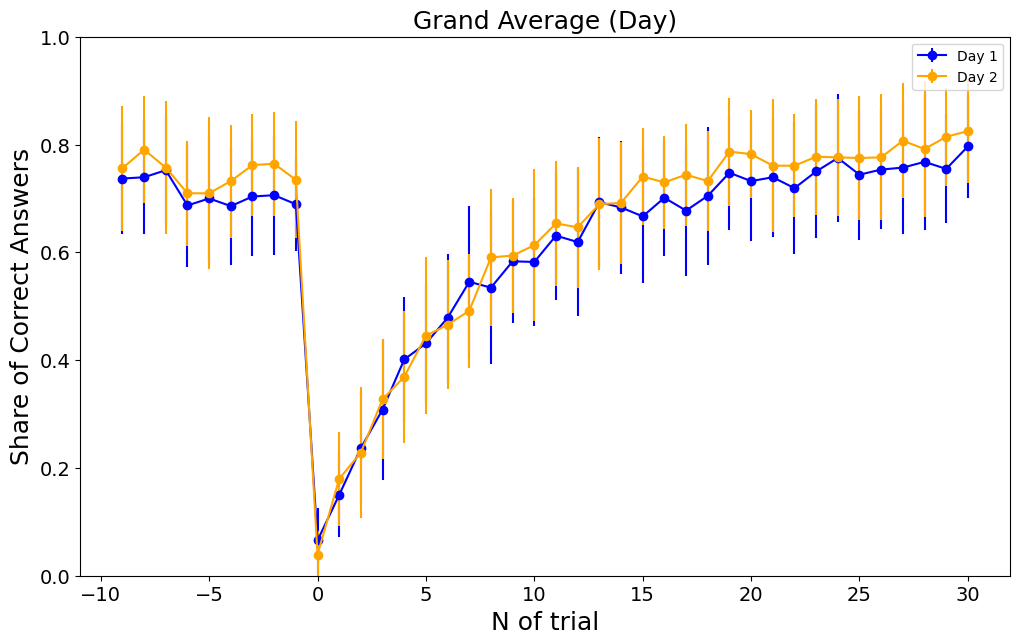


*Figure S6.* Average performance around the combination switch for behavioral data (day 1 versus day 2). The performance does not differ between the conditions. Each dot represents the mean and standard deviation of the share of correct answers averaged across all participants.


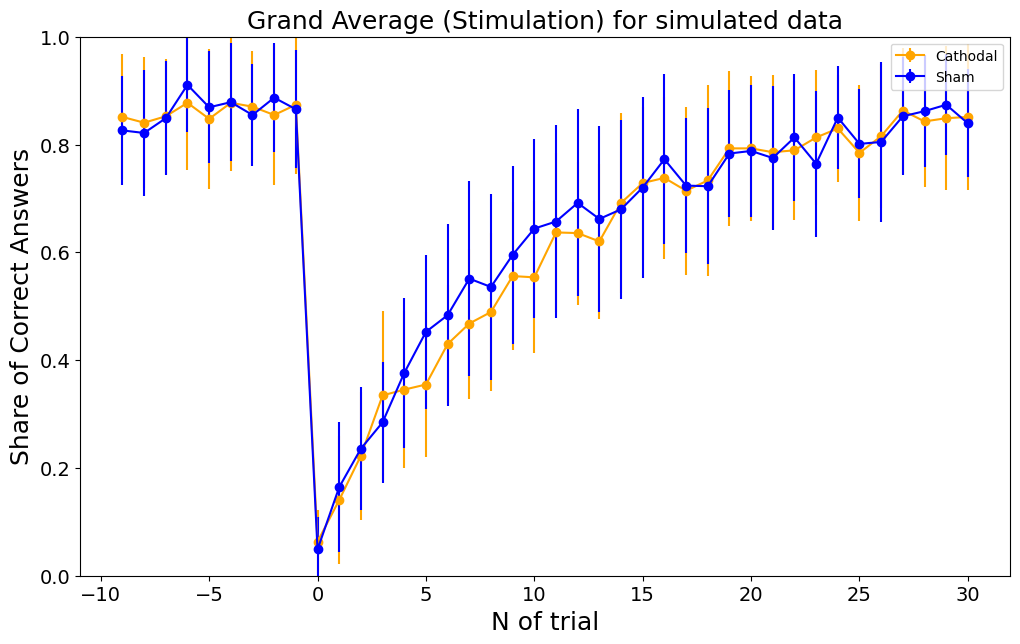


*Figure S7.* Average performance around the combination switch for simulated data (cathodal versus sham conditions). The performance does not differ between the conditions. Each dot represents the mean and standard deviation of the share of correct answers averaged across all participants.


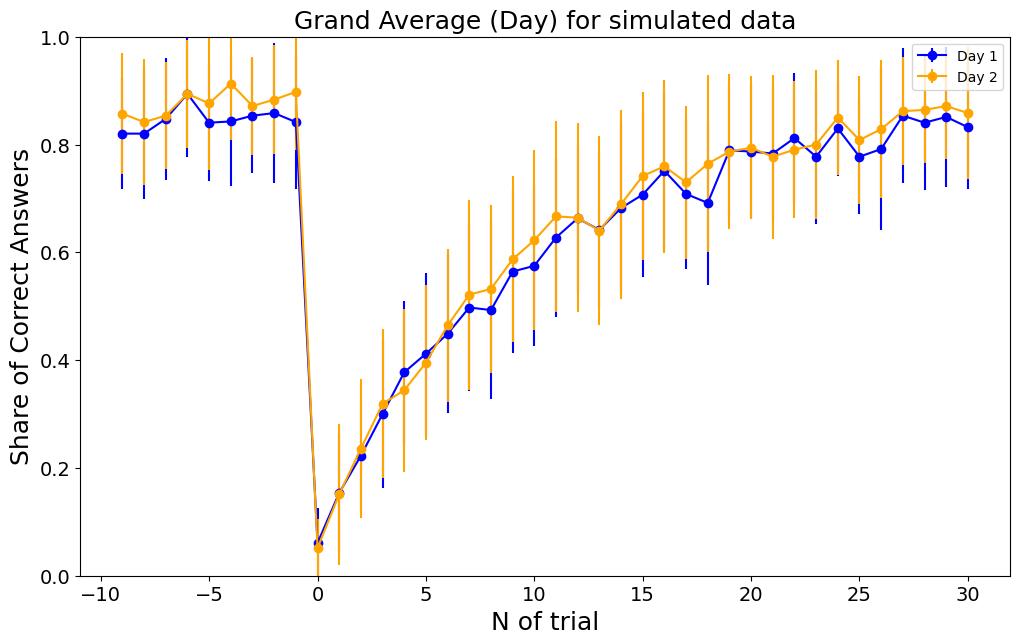


*Figure S8.* Average performance around the combination switch for simulated data (day 1 versus day 2). The performance does not differ between the conditions. Each dot represents the mean and standard deviation of the share of correct answers averaged across all participants.

***Table S1***

*Comparison of ground truth and recovered parameters on simulated data (based on parameters from Domenech et al., 2020 and confirmation bias from Donoso et al., 2014).*

|  | | N | | α | | π | | β | | ε | | τ | | θ | η | LL | |
| --- | --- | --- | --- | --- | --- | --- | --- | --- | --- | --- | --- | --- | --- | --- | --- | --- | --- |
|  | Ground truth | | 2 | | 0.55 | | 1.51 | | 66.6 | | 0.12 | | 0.13 | 0.82 | 0.57 | -480.84 |  |
|  | Recovered | | 2 | | 0.56 | | 2.72 | | 54.4 | | 0.13 | | 0.12 | 0.91 | 0.58 | -477.33 |  |
|  | Ground truth | | 3 | | 0.32 | | 1.19 | | 62.0 | | 0.16 | | 0.09 | 0.72 | 0.50 | -530.83 |  |
|  | Recovered | | 5 | | 0.28 | | 1.13 | | 60.3 | | 0.15 | | 0.10 | 0.70 | 0.46 | -529.24 |  |
|  | Ground truth | | 3 | | 0.48 | | 1.65 | | 117.0 | | 0.17 | | 0.11 | 0.62 | 0.25 | -551.74 |  |
|  | Recovered | | 5 | | 0.63 | | 1.28 | | 155.9 | | 0.17 | | 0.15 | 0.04 | 0.50 | -546.50 |  |
|  | Ground truth | | 2 | | 0.28 | | 1.14 | | 107.0 | | 0.04 | | 0.14 | 0.47 | 0.11 | -295.10 |  |
|  | Recovered | | 2 | | 0.28 | | 1.10 | | 83.7 | | 0.04 | | 0.11 | 0.46 | 0.11 | -290.16 |  |
|  | Ground truth | | 2 | | 0.40 | | 1.45 | | 153.3 | | 0.14 | | 0.12 | 0.95 | 0.25 | -506.34 |  |
|  | Recovered | | 2 | | 0.45 | | 1.06 | | 88.9 | | 0.14 | | 0.11 | 1.0 | 0.37 | -503.39 |  |
|  | Ground truth | | 2 | | 0.19 | | 2.44 | | 96.0 | | 0.11 | | 0.12 | 0.55 | 0.33 | -471.05 |  |
|  | Recovered | | 2 | | 0.18 | | 2.61 | | 82.8 | | 0.11 | | 0.12 | 0.68 | 0.32 | -467.38 |  |

*Notes.* N – Monitoring buffer capacity, α – learning rate in reinforcement learning (RL), π – Bayesian prior, β – inverse temperature in RL, ε - 'lapse' rate of softmax (i.e., noise), τ – perceived volatility, θ – confirmation bias, η - recollection entropy, LL – loglikelihood of choice probabilities in simulated data.

***Table S2***

*Descriptive statistics for behavioral data*

|  | Correct answers  M ± SD | Correct answers  Min | Correct answers  Max |
| --- | --- | --- | --- |
| Day 1 (N = 42) | 686.26 ± 73.07 | 480 | 846 |
| Day 2 (N = 42) | 723.17 ± 60.29 | 571 | 831 |
| Verum stimulation (N = 42) | 701.81 ± 65.08 | 573 | 846 |
| Sham stimulation (N = 42) | 707.62 ± 73.62 | 480 | 831 |
| All data (N = 84) | 704.71 ± 69.12 | 480 | 846 |

***Table S3***

*Descriptive statistics for the number of exploratory trials (with parameters fitted to the behavioral data)*

|  | Exploratory trials  M ± SD | Exploratory trials  Min | Exploratory trials  Max |
| --- | --- | --- | --- |
| Day 1 (N = 42) | 262.88±152.63 | 29 | 617 |
| Day 2 (N = 42) | 233.88±158.40 | 12 | 558 |
| Verum stimulation (N = 42) | 254.45±164.62 | 29 | 617 |
| Sham stimulation (N = 42) | 242.31±147.11 | 12 | 583 |
| All data (N = 84) | 248.38±155.29 | 12 | 617 |

***Table S4***

*Estimated parameters for the behavioral data (M±SD, [min, max]).*

|  | N | α | π | β | ε | τ | θ | η | LL |
| --- | --- | --- | --- | --- | --- | --- | --- | --- | --- |
| All data | 3.7±1.6  [1, 6] | 0.42±0.23  [0.01, 1.0] | 2.08±0.69  [1.0, 3.0] | 22.8±48.1  [3.1, 200] | 0.11±0.07  [0.00001, 0.34] | 0.20±0.12  [0.001, 0.37] | 0.63±0.37  [0, 1] | 0.18±0.18  [0, 0.87] | -714.5±145.3  [-1022.3, -392.4] |
| Sham | 3.3±1.6  [1, 6] | 0.41±0.23  [0.01, 1.0] | 2.20±0.71  [1.0, 3.0] | 27.3±56.2  [4.0, 200] | 0.12±0.07  [0.00001, 0.29] | 0.20±0.12  [0.01, 0.37] | 0.62±0.37  [0, 1] | 0.18±0.18  [0, 0.87] | -710.7±144.4  [-1022.3, -392.4] |
| Verum | 4.0±1.6  [1, 6] | 0.43±0.24  [0.01, 0.90] | 1.96±0.67  [1.0, 3.0] | 18.3±38.4  [3.1, 179.4] | 0.10±0.06  [0.00001, 0.34] | 0.19±0.12  [0.01, 0.35] | 0.63±0.38  [0, 1] | 0.19±0.19  [0, 0.72] | -718.3±147.9  [-1005.0, -411.8] |
| Day 1 | 3.6±1.6  [1, 6] | 0.49±0.25  [0.01, 1.0] | 2.05±0.73  [1.0, 3.0] | 14.0±29.4  [3.1, 148.2] | 012±0.07  [0.00001, 0.34] | 0.21±0.12  [0.01, 0.37] | 0.61±0.38  [0, 1] | 0.22±0.21  [0, 0.87] | -754.9±145.4  [-1022.3, -411.8] |
| Day 2 | 3.7±1.7  [1, 6] | 0.35±0.20  [0.01, 0.72] | 2.11±0.66  [1.0, 3.0] | 31.7±60.5  [3.6, 200] | 0.10±0.06  [0.00001, 0.29] | 0.18±0.13  [0.01, 0.37] | 0.65±0.36  [0, 1] | 0.15±0.14  [0, 0.66] | -674.2±135.3  [-978.3, -392.4] |

*Notes.* N – Monitoring buffer capacity, α – learning rate in reinforcement learning (RL), π – Bayesian prior, β – inverse temperature in RL, ε - 'lapse' rate of softmax (i.e., noise), τ – perceived volatility, θ – confirmation bias, η - recollection entropy, LL – loglikelihood of choice probabilities in behavioral data. In some cases, very small numbers were rounded to 0. See analysis code on OSF for exact numbers.
